# Supplementary material for: Performance of neural network basecalling tools for Oxford Nanopore sequencing
Source: Genome Biol. 2019 Jun 24;20:129. doi: 10.1186/s13059-019-1727-y (PMC6591954; doi:10.1186/s13059-019-1727-y)
Supplement: Supplementary file 1 — Supplementary figures. This file contains supplementary figures S1–S6. (PDF 3513 MB) [file 13059_2019_1727_MOESM1_ESM.pdf]

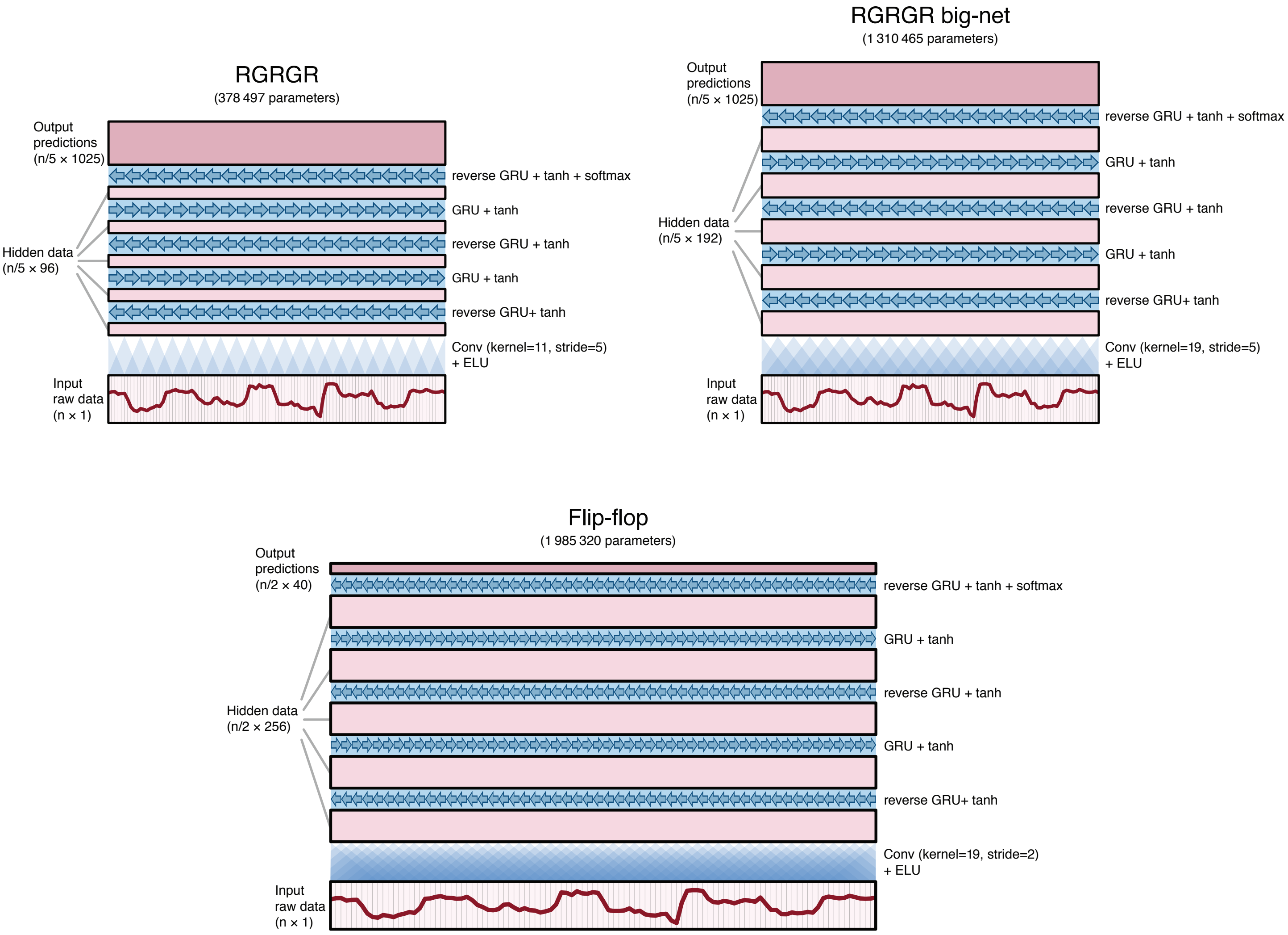

Fig S1 ONT neural network architectures. The RGRGR network (top-left) is used in Albacore, Guppy and Scrappie raw, and it was used to train the custom-Kp model. The big-net (top-right) is a modification of the RGRGR network, with a wider convolution (kernel of 19 vs 11) and larger hidden data (192 filters vs 96). For both networks, the convolutional layer reduces the data length (n) by a factor of five (i.e. there are five raw data values per predicted state). There are 1025 possible predicted values: 1024 for each possible 5-mer plus an additional `stay' state. The flip-flop model (bottom) is a newer architecture introduced in Flappie and available in recent versions of Guppy. It features a wide convolution (kernel of 19), a smaller stride that only reduces the data length by a factor of 2, large hidden data (256 filters) and a CTC decoder.

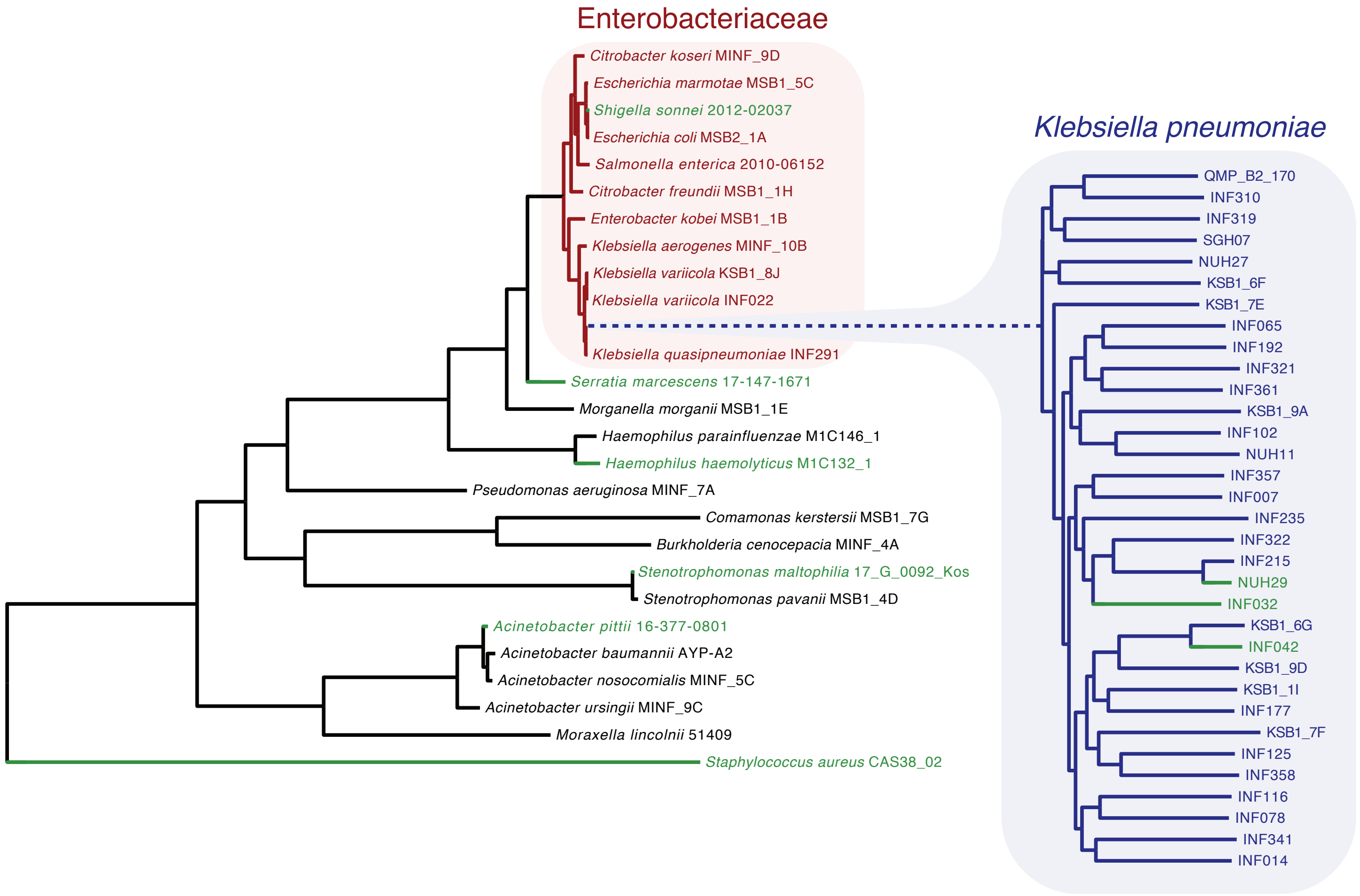

Fig S2 Genome phylogeny. Tree of the genomes used for custom Sloika training. All isolates are from the Proteobacteria phylum, with an emphasis on the Enterobacteriaceae family (red) and *Klebsiella pneumoniae* (blue). The genomes used to test basecallers/models are also included in this tree in green, but were not used to make training data.

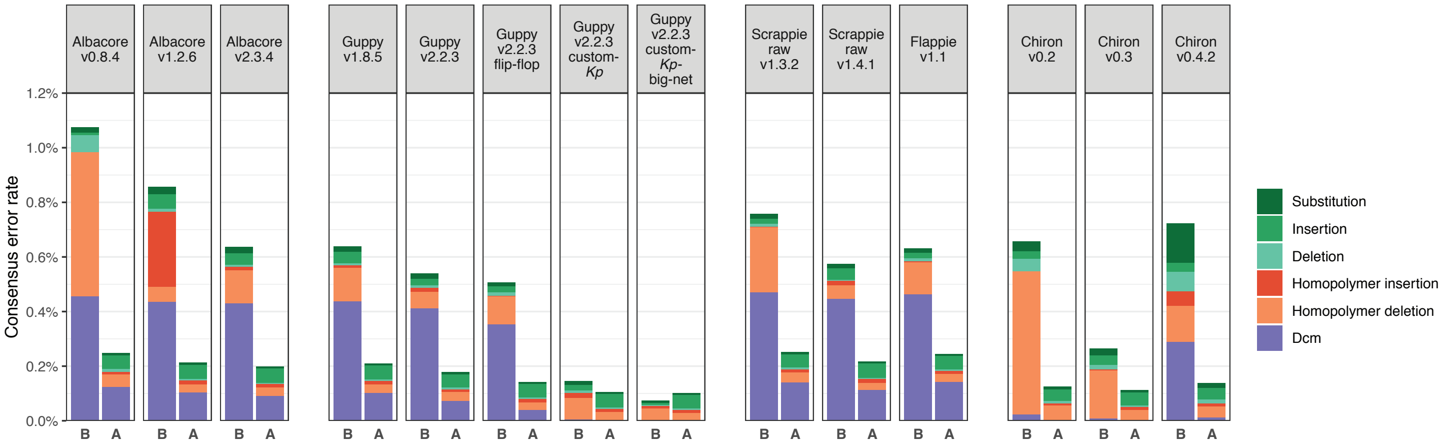

Fig S3 Nanopolish error details. Consensus errors per basecaller, broken down by type (same types as Fig 3), before (B) and after (A) Nanopolish. Non-Dcm errors are relatively occur at a similar rate in each post-Nanopolish assembly, but the number of Dcm errors depends on their prevalence in the pre-Nanopolish assembly.

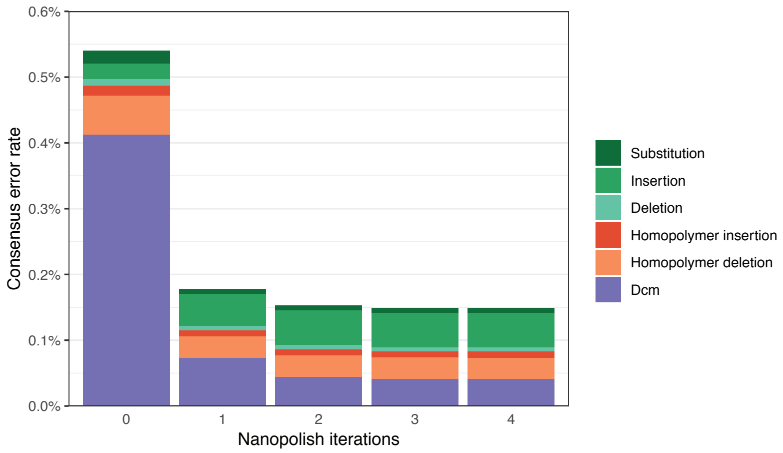

Fig S4 Nanopolish iterations. Consensus errors with repeated iterations of Nanopolish, using the Guppy v2.2.3 dataset. Accuracy improves only slightly with successive iterations, with most of the improvement coming from Dcm errors.

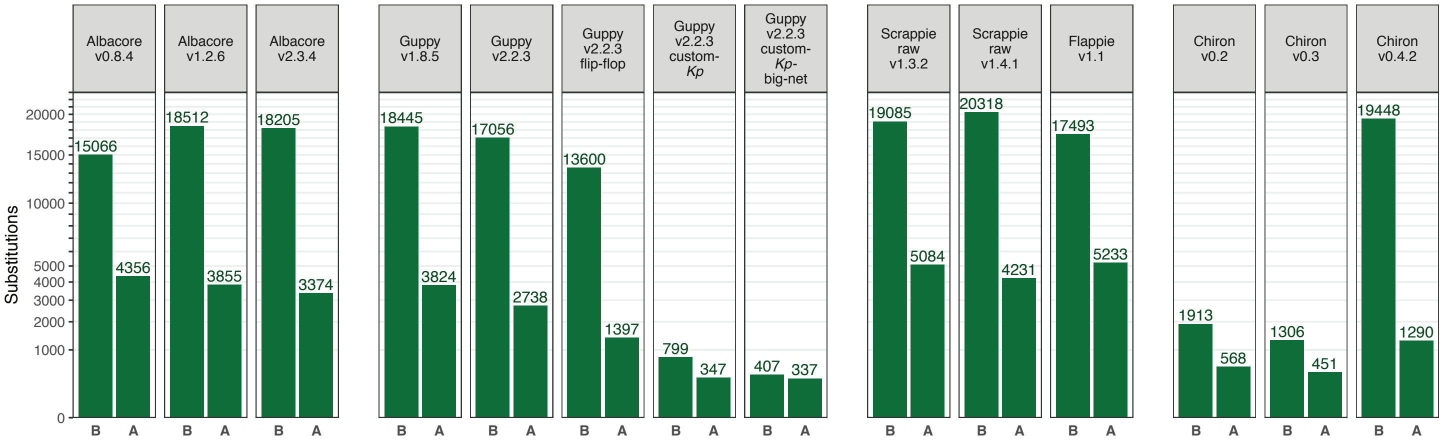

Fig S5 Substitution counts. Number of consensus sequence substitution errors per basecaller, before (B) and after (A) Nanopolish. All substitution types (both Dcm-related and non-Dcm-related) are counted.

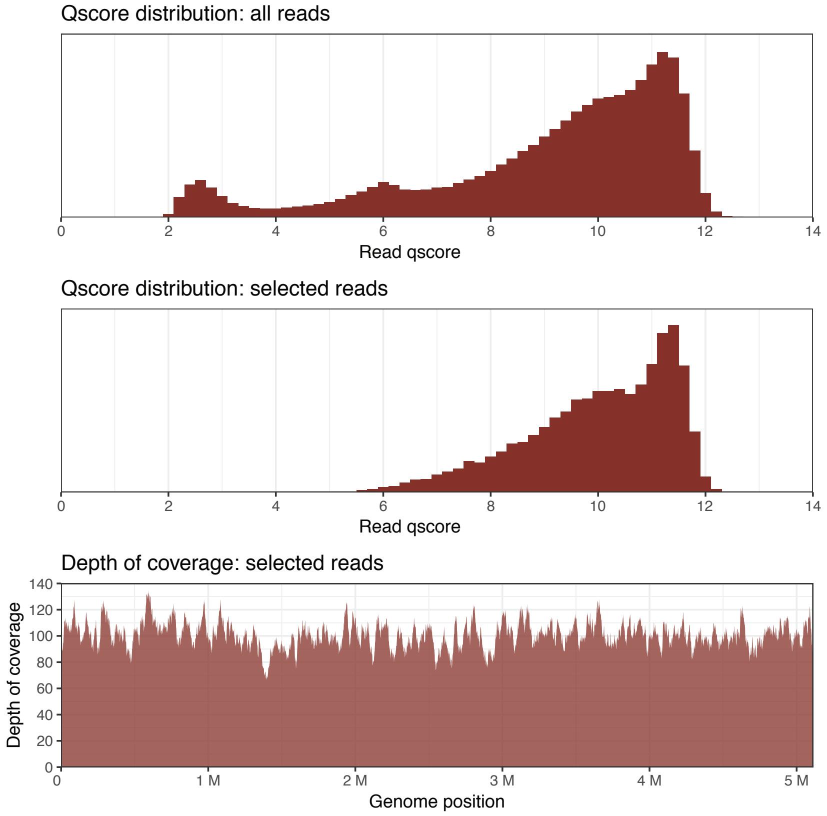

Fig S6 Qscore distributions and depth of coverage. Qscore distributions (according to Guppy v1.6.0) for the entire barcoded MinION run (top) and the selected INF032 reads (middle), and the depth of coverage across the reference genome for the selected reads (bottom).
