# Supplementary material for: Performance of neural network basecalling tools for Oxford Nanopore sequencing
Source: Genome Biol. 2019 Jun 24;20:129. doi: 10.1186/s13059-019-1727-y (PMC6591954; doi:10.1186/s13059-019-1727-y)
Supplement: Supplementary file 3 — Review history. This document contains the peer review history for the manuscript. (DOCX 26 kb) [file 13059_2019_1727_MOESM3_ESM.docx]

Review History

**First round of review**

**Comments to author and authors’ point-by-point responses to the reviewers’ comments:**

**Reviewer 1**

1. In the section of default model performance, the authors simply described which tools performed better or worse. It would be better to add brief discussions about the possible reasons. For example, was it caused by the difference on model architecture, or the ways that the models were training?

*We have revised the text in the paragraph describing Albacore’s results to be more explicit about the reasons for its accuracy improvements.*

*Previously: Albacore’s development history shows two distinct improvements in both read and consensus accuracy: in April 2017 (version 1.0.1) and August 2017 (version 2.0.1) (Fig 1). The first corresponds to the addition of a transducer…*

*Currently: Albacore’s history contained two major developments which resulted in distinct improvements in both read and consensus accuracy: in April 2017 (version 1.0.1) and August 2017 (version 2.0.1) (Fig 1). The first was the addition of a transducer…*

*We also made an addition to the paragraph describing Scrappie’s performance:*

*Previously: Scrappie events was the worst-performing basecaller tested.*

*Currently: Scrappie events was the worst-performing basecaller tested, likely due to its use of an outmoded event-segmentation pre-processing step.*

*Reasons for Chiron’s performance are addressed in the following comment.*

2. In Fig. 1, Chiron performed generally worse than Albacore, but the version between Oct. 2017 and Jan. 2018 had much better performance on consensus accuracy. What was the possible reason? Chiron v0.3 performed well on consensus accuracy because its model was trained on sequencing reads from E. coli – a close relative of Klebsiella pneumoniae.

*To clarify this, we have revised the text in the ‘Default model performance’ section: Previously: Relative to ONT’s basecallers, Chiron performed poorly on read accuracy (Fig 1). However, Chiron v0.3 had the highest consensus accuracy (Q25.9) of all tested basecallers using their default models. The latest version, v0.4.2, did not perform as well on our benchmarking set (Q7.7 read accuracy and Q21.4 consensus accuracy) due to its use of human DNA training data (see consensus error profiles below).*

*Currently: Relative to ONT’s basecallers, Chiron performed poorly on read accuracy (Fig 1). Chiron v0.3 had the highest consensus accuracy (Q25.9) of all tested basecallers using their default models, but the latest version (v0.4.2) did not perform as well on our benchmarking set (Q7.7 read accuracy and Q21.4 consensus accuracy). This variation in accuracy for different versions of Chiron is explained by the taxonomy of the training data used to produce their default models (E. coli vs human, see Consensus error profiles).*

3. The model architecture of RGRGR and RGRGR big-net were illustrated in Fig. S5. It is better to give some brief introductions about "flip-flop".

*With ONT’s recent release of Taiyaki (a tool for training flip-flop models), the flip-flop architecture is now more accessible.*

*We have thus added an illustration of this model to network architecture figure (now numbered S1 due to its earlier reference in the text) and the following explanation to the results section: However, the flip-flop model’s accuracy comes at the cost of speed performance – it is considerably slower than the default Guppy model, likely due to its larger size and complexity (Fig S1).*

4. The read identity and consensus accuracy were defined as the number of matching bases in the alignment divided by the total alignment length including insertions and deletions. Was the proportion of the aligned fragment taken into consideration? A read could also get a high identity when only a small fragment was aligned.

Yes, we did consider the alignment length and have revised the text in the ‘Read and consensus accuracy’ section to clarify this point:

*Previously: Unaligned reads were given an identity of 0%.*

*Currently: If less than half of a read aligned to the reference, it was deemed unaligned and given an identity of 0%.*

5. I did not find the commands for Sloika on custom model training from github. Could the authors provide them? It would be helpful to the users who also need custom model training in their applications.

*We have now added the Sloika commands to the GitHub repository (github.com/rrwick/Basecalling-comparison) for others to use when producing their own custom-trained models.*

6. In addition to bacteria data, the performance on mouse and human data is also very important to the community. Moreover, there would be an increasing tendency for applying ONT to RNA sequencing. The authors should provide brief discussions over these points.

*We have added the following text to the conclusion section to cover these points: For example, a model trained on native human DNA may also perform well on native mouse DNA and vice versa, as CpG methylation is common to both species [11]. The benefits of custom training may further extend to direct RNA sequencing, where base modifications can be more extensive than in DNA [12, 13].*

**Reviewer 2**

1. Bibliographical reference are non-existent, which makes it impossible to verify the correctness of the citations to previous studies.

*The paper does have a references section (of course!) so we are not sure what went wrong. Perhaps a problem with the Editorial Manager or LaTeX to pdf conversion? Hopefully the next version you receive with our recent edits will not suffer the same problem.*

2. Adding more info about the datasets in the Results and Discussion section, "default model performance" subsection, (e.g., genome size, average/median read length, depth of coverage, etc) can be beneficial to the read to get a better understanding of the underlying datasets when reading about the quality of the described basecallers' results.

*We have added the following paragraph to the beginning of the Results and discussion section, to offer better context in which readers can interpret the results: Our primary benchmarking read set for assessing basecaller performance consisted of 15154 whole genome sequencing reads from an isolate of Klebsiella pneumoniae (see methods). These reads ranged from 22–134 kbp in length (N50=37 kbp) and were ∼550 Mbp in total size, equating to 100× read depth over the 5.5 Mbp K. pneumoniae chromosome.*

3. line 158: adding information about relative percentage of Dcm-methylations sites consensus errors relative to all error (in addition to the reference) will be beneficial to the reader.

*We have revised the text to include the proportion of Dcm-related errors both in terms of total errors and the reference: Previously: …and these made up a large proportion of total consensus errors (~0.4% error relative to the reference). Currently: …and these made up a large proportion of consensus errors: for current versions of ONT basecallers ∼70% of the errors were in Dcm motifs and they created ∼0.4% error relative to the reference.*

4. lines 260-264: quantification of "unrealistic large amount of RAM" is suggested; evidence that reduction in chunk size/batch number does not significantly affect resulting trained model is lacking, and either a reference to a previous study, or additional information is required.

*Our modifications to Sloika did not change the size of the chunks or the training batches. They were simply workarounds to avoid keeping our entire training set in memory at once. Our use of ‘smaller batches’ in that passage was misleading, as we did not use ‘batches’ in the usual sense for neural network training. We have revised that section to include more details on RAM usage and clarify our modifications to Sloika:*

*Previously: Using Sloika with this entire training set would have required an unrealistically large amount of RAM, so we produced a fork of Sloika (github.com/rrwick/sloika) modified to load training data in smaller batches.*

*Currently: Using Sloika with this entire training set would have required hundreds of gigabytes of RAM, so we produced a fork of Sloika (github.com/rrwick/sloika) modified to load a random subset of the training data at periodic intervals. By only training on 5% of the data at a time and reloading a new 5% every 250 training batches, we were able to use the entirety of our training data while keeping RAM usage under 10 GB.*

5. lines 300-302: was the mean ~100x read depth coverage uniform across the genome after read filtration?

*Yes, the post-filtration coverage was relatively uniform. To illustrate this, we have added a depth-of-coverage plot to Figure S6 (which also shows the qscore distributions before and after filtering) and made the following addition to the text: Previously: …was chosen because it reduced the dataset to approximately 100× mean read depth for the INF032 genome. Currently: …was chosen because it reduced the dataset to approximately 100× mean read depth for the INF032 genome while maintaining even coverage (Fig S6).*

6. "Read and consensus accuracy" subsection: clarification that the INF032 reference with which both the read accuracy and the consensus accuracy are measured is sample-specific and obtained from the high-quality Illumina based assembly (described on lines 284-290).

*To clarify, we have added the following sentence to that section: This reference was used as the ground truth sequence when assessing read and consensus accuracy for each basecaller (see below).*

7. lines 347-349: quantification of the "more accurate" is suggested.

*We have revised and expanded on that section to quantify the improvement in consensus accuracy achieved with the iterative assembly approach:*

*Previously: We used 10 iterations, each of which resulted in a slightly different assembly. These ten assemblies were then used as the ‘reads’ for a final Rebaler assembly, giving a more accurate result than any of the 10 input iterations.*

*Currently: We used 10 iterations, each resulting in a slightly different assembly which were then used as the ‘reads’ for a final Rebaler assembly. This iterative approach was able to reduce assembly errors by about 20%: individual Rebaler assemblies of the Guppy v2.2.3 reads had a consensus accuracy of 99.33% (Q21.74) but the final iterative assembly had an accuracy of 99.47% (Q22.76).*

8. lines 352-353: some comment about distribution of the consensus accuracy across considered 1-kbp pieces is suggested.

*We have added the following sentence to the methods paragraph on read accuracy: Read accuracy distributions had a considerable amount of variance, ranging from ∼95% identity for the best reads down to ∼60%, below which reads were too noisy to be aligned by minimap2. …and the following sentence to the methods paragraph on consensus accuracy: The distributions of identities for assembly pieces were narrow and had standard deviations of which We have revised that sentence for clarity:*

*Previously: ONT's progress on this front is evident in the performance of Albacore, whose consensus accuracy improvements over time have mostly come from…*

*Currently: ONT's progress on this front is evident in the performance of Albacore, for which consensus accuracy improvements over time have mostly come from…*

**Second round of review**

**Reviewer 1**

1.The accuracy that is discussed in this benchmark is merely the alignment identity of the alignable regions of read. It does surely overestimate the real accuracy of the data. More importantly, some software might be implemented to only output selective subset of the data where low-quality or non-alignable reads might be ignored so as to have "high-quality" output. It would be a more fair benchmark to include a specific section discussing the alignable fractions of the output from different software so that the readers could have a better understanding of the whole picture.

*Firstly, it is important to note that our goal is not to produce some measure of overall accuracy/call-ability of all signal generated by a Nanopore run, which will be affected by the amount of non-callable noise in the raw reads and is thus a function of the device not the basecaller. Rather our goal is to compare the performance (accuracy and speed) of basecalling software on a common set of call-able (alignable) input reads. The input reads we used to assess basecalling accuracy were selected based on their ability to align to the reference genome, and the vast majority of basecalled reads outputted by the tested software were found to be alignable across their entire sequence (median per-read alignable fraction >99.5% in all cases, with most of the unaligned regions due to sequencing adapters and barcode sequences). In the rare instances where an output read was not alignable along >50% of its length (<6% for all callers, now reported in the supplementary table, ‘Benchmarking set results’ in File S1), we assigned it an identity of 0% along its full length. Each basecaller outputted the full set of reads, with one exception: the oldest version of Albacore failed to output 438 (3%) of the input reads, and for these we assigned an identity of 0% along their full length. Our reported read-level accuracy value for each basecaller (the median value across all reads) therefore provides a fair and comparable performance metric that is not biased by alignable-vs-unalignable regions and is not misled by differences in how the basecallers might handle low-quality reads as suggested by the reviewer.*

*To clarify these points to readers, we have added columns to our supplementary table (‘Benchmarking set results’ in File S1) reporting the total number of basecalled reads, the proportion of unaligned reads and the per-read alignable fractions and for each basecaller. We have also expanded upon these concepts in the manuscript text (pages 13–14):*

*Previously:	To assess read accuracy, we aligned each basecalled read set to the reference INF032 genome using minimap2 [18] (v2.12). Each read’s identity was defined as the number of matching bases in its alignment divided by the total alignment length including insertions and deletions, a.k.a. the ‘BLAST identity’ (lh3.github.io/2018/11/25/on-the-definition-of-sequence-identity). If less than half of a read aligned to the reference, it was deemed unaligned and given an identity of 0%. Read accuracy distributions had a considerable amount of variance, ranging from ∼95% identity for the best reads down to ∼60%, below which reads were too noisy to be aligned by minimap2. We therefore defined each basecaller’s overall read accuracy as the median identity of the reads in the set.*

*Currently:	To assess read accuracy, we aligned each basecalled read set to the reference INF032 genome using minimap2 [18] (v2.12). Each read’s identity was defined as the number of matching bases in its alignment divided by the total alignment length including insertions and deletions, a.k.a. the ‘BLAST identity’ (lh3.github.io/2018/11/25/on-the-definition-of-sequence-identity). Our read identity values were therefore based only on the fraction of each read which aligned to the reference (‘per-read aligned fraction’), though if less than half of a read aligned it was given an identity of 0% (<6% of reads for all basecallers, see File S1). While this approach could overestimate the identity of each read (which may consist of both aligned and unaligned regions), our read selection process (only using reads with a long alignment to the reference, see above) resulted in reads with few unaligned regions: for our benchmarking set, each basecaller had a median per-read aligned fraction of >99.5% (File S1), with most of the unaligned regions due to sequencing adapters and barcode sequences. Basing read identity on aligned regions should therefore provide a valid assessment of overall read identity for our test sets, (although note this same approach may not be appropriate for other read sets where quality control has not been performed).*

*Another possible concern is that some basecallers might only output a selective subset of the data, i.e. instead of producing basecalled sequence for all reads, they might only output the higher quality reads, resulting in an inflated average identity. To guard against this possibility, any reads missing from the output were given an identity of 0%. However, of all basecallers and versions, only Albacore v0.7.5 (the oldest version tested) failed to output all 15154 reads in our benchmarking set (3% missing reads, see File S1). The distribution of per-read identities for each basecaller was broad, with most reads in the range of 70% to 95%. We thus defined each basecaller’s overall read accuracy as the median identity across all reads in the set (counting the small fraction that were not reported or not alignable along >50% of the read length as 0% identity).*

2.Human or mouse data should be included as they are of highest biomedical impact and interest (question #6). I understand it would be very lengthy and take a lot of work to include human or mouse data throughout the whole manuscript, I think at least there should be an extra section to include some results of human or mouse data, showing some consistency of the results.

*While we agree that results using human DNA would be of interest, unfortunately this is not currently possible. The heterozygous nature and high repetitive content of human DNA result in errors in hybrid assemblies, so no confident ground-truth sequence is available for assessing basecaller accuracy. This is why ONT testing protocols utilise bacteriophage DNA, and the published basecallers have utilised haploid bacterial DNA for their assessment of consensus accuracy. Our use of bacterial DNA for benchmarking basecalling accuracy is thus in line with the field standard.*

*Importantly, we know of no reason to suspect that the relative performance of basecallers would be different when using human DNA (as opposed to using different training data, which our study suggests would improve performance when comparing the same custom-trainable basecaller using two different models, but this is not currently possible to test directly due to the lack of ground truth for human DNA with which to train and test such models).*

*Therefore, we cannot add an extra section showing results on human or mouse data. Instead, we have added the following paragraph to the manuscript to more clearly explain and justify our reasons for limiting our analyses to bacterial DNA (pages 12–13):*

*Despite the research interest in human DNA, we chose to limit our read sets to bacterial samples because they allow for a more confident ‘ground truth’ reference sequence against which to calculate accuracy than do complex eukaryote genomes. Bacterial genomes are haploid and have relatively low repetitive content (due in part to >85% of sequence being protein coding), facilitating complete assembly and accurate Illumina-read polishing against which to calculate ONT basecalling accuracy. A recent study on de novo assembly of human ONT reads [6] found a 0.04% error rate between an Illumina-polished assembly and reference sequence. This level of uncertainty in the reference would make a Q50 consensus appear to be Q34, so estimates of consensus sequence accuracy could only provide a lower bound on the true value. Additionally, the small sizes of bacterial genomes make computational tasks more tractable and facilitate higher read depths. For these reasons, bacterial and phage DNA have been the standard in ONT accuracy testing and basecaller development, with previous studies either exclusively using bacterial DNA [4] or limiting human DNA to read-level analyses (i.e. excluding consensus-level analyses) [3, 5].*
